# Clinical and Biological Data in Patients with Pancreatic Cancer vs. Chronic Pancreatitis—A Single Center Comparative Analysis

**DOI:** 10.3390/diagnostics13030369

**Published:** 2023-01-19

**Authors:** Gina Gheorghe, Vlad Alexandru Ionescu, Horatiu Moldovan, Camelia Cristina Diaconu

**Affiliations:** 1 Faculty of Medicine, University of Medicine and Pharmacy Carol Davila Bucharest, 050474 Bucharest, Romania; 2 Gastroenterology Department, Clinical Emergency Hospital of Bucharest, 105402 Bucharest, Romania; 3 Department of Cardiovascular Surgery, Clinical Emergency Hospital of Bucharest, 105402 Bucharest, Romania; 4 Medical Sciences Section, Academy of Romanian Scientists, 050085 Bucharest, Romania; 5 Internal Medicine Department, Clinical Emergency Hospital of Bucharest, 105402 Bucharest, Romania

**Keywords:** pancreatic cancer, chronic pancreatitis, risk factors, late diagnosis

## Abstract

Introduction: In some patients with chronic pancreatitis, the diagnosis of pancreatic cancer can be missed. The objective of the study was to identify clinical and paraclinical data with statistical significance in the differential diagnosis between chronic pancreatitis and pancreatic cancer. Materials and Methods: We conducted a retrospective, observational study on a cohort of 120 patients hospitalized over 3 years. The patients were equally distributed in two groups: group A, with 60 patients with pancreatic cancer, and group B, with 60 patients with chronic pancreatitis. The statistical analysis was carried out by using the R program. Results. The comparative analysis of pancreatic cancer vs. chronic pancreatitis revealed a stronger link between pancreatic cancer, female gender (*p* = 0.001) and age over 60 years (*p* < 0.001). Patients with pancreatic cancer had higher serum values of aspartate aminotransferase (*p* 0.005), alanine aminotransferase (*p* 0.006), total bilirubin (*p* < 0.001), direct bilirubin (*p* < 0.001), alkaline phosphatase (*p* 0.030), C-reactive protein (*p* = 0.049) and uric acid (*p* 0.001), while patients with chronic pancreatitis presented slightly higher values of amylase (*p* 0.020) and lipase (*p* 0.029). Conclusions: Female gender, advanced age, elevated aminotransferases, cholestasis markers and uric acid were associated with a higher probability of pancreatic cancer.

## 1. Introduction

Pancreatic cancer is a condition with a low prevalence but a high mortality [1]. The incidence of pancreatic cancer worldwide is approximately 1.6%, and the prevalence of pancreatic cancer in the United States of America (USA) is 9 cases per 100,000 inhabitants in all age groups, increasing to 68 cases per 100,000 inhabitants in the group more than 55 years old [1,2]. Currently, pancreatic cancer ranks 11th worldwide in the ranking of newly diagnosed cases of cancer (with 495,773 new cases reported in 2020) and 7th in the ranking of deaths caused by cancer (with 466,003 deaths reported in 2020) [3,4]. According to data from 2020, pancreatic cancer represents 3% of all newly diagnosed cancer cases and approximately 8% of all cancer deaths in the USA [5]. Additionally, an increase in the incidence of pancreatic cancer is anticipated in the next decade [6].

Despite the progress in the management of patients with different types of malignant neoplastic diseases and the most innovative techniques, the survival rate of patients with pancreatic cancer has remained almost unchanged over the last decades [7,8,9,10]. The 5-year survival rate of patients with this malignancy is approximately 10% [11,12]. The poor prognosis is due to the aggressive behavior of the disease from the biological point of view, the non-specific symptomatology until advanced stages, the absence of sensitive and specific methods for an early diagnosis and also the poor response to oncological treatments [11,13]. The only therapeutic approach with curative potential remains surgical resection [11,14]. However, only 10–20% of cases are amenable to surgical resection, with approximately 75% of pancreatic cancer patients being diagnosed in stages III–IV [11,12,13,14,15].

**Figure 1:**
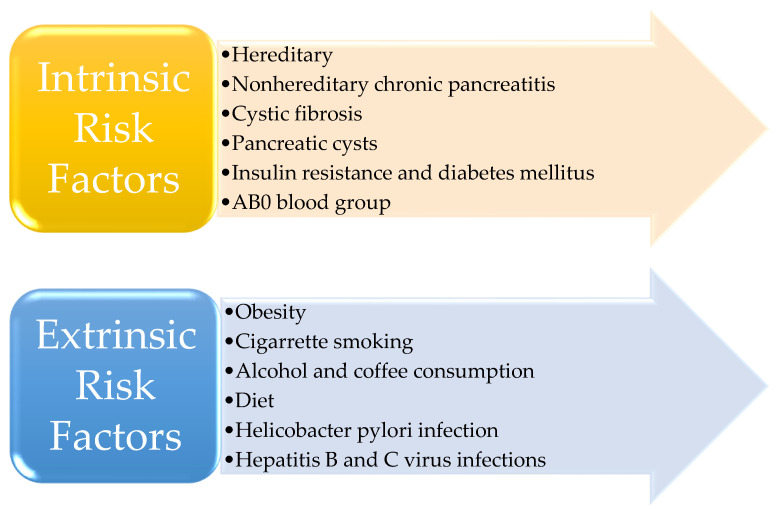
The risk factors for pancreatic cancer.

The risk factors involved in the occurrence of pancreatic cancer are divided into two major categories: intrinsic risk factors and extrinsic risk factors (Figure 1) [16,17,18,19].

Chronic pancreatitis is a potential risk factor implicated in the occurrence of pancreatic cancer [16,17]. This condition is characterized by a chronic inflammatory process of the pancreatic parenchyma, which ultimately leads to irreversible fibrosis and pancreatic exocrine and endocrine insufficiency [20]. The global incidence of chronic pancreatitis is 9.62 cases per 100,000 inhabitants [21]. The literature highlights an increasing tendency in the incidence of this condition [22,23,24]. For instance, Olesen et al. report an increase in the prevalence of chronic pancreatitis in the Danish population from 126.6 cases per 100,000 inhabitants in 1996 to 153.9 cases per 100,000 inhabitants in 2016 [23]. The same study also emphasizes an increase in the median age at the diagnosis of this condition from 52.1 years to 60 years during the study period (1994–2018) [23].

Kirkegard et al. report that 2 years after establishing the diagnosis of chronic pancreatitis, the relative risk of evolution towards pancreatic cancer is around 16.16% [25]. The same authors highlight a decrease in the risk of developing pancreatic cancer with a prolonged period from the diagnosis of chronic pancreatitis [20]. Hence, at 5 years, the risk of pancreatic cancer among patients with chronic pancreatitis was 7.9%, and at 9 years it was 3.53% [25]. Another study that followed patients with chronic pancreatitis over a longer period reported a cumulative risk of evolution to pancreatic cancer of 1.8% at 10 years and 4% at 20 years, independent of the type of pancreatitis [26].

Genetic changes, particularly the K-Ras mutation, have the greatest impact on the progression of pancreatic precursor lesions to pancreatic ductal adenocarcinoma [27]. Additionally, various immune suppression mechanisms may occur in the tumor environment to prevent effective antitumor immunity [27]. Tumor cells are thought to evade immune responses by avoiding checkpoint control, which, by blocking the inhibitor activity of T-cell-mediated immune responses, improves the immune system’s responses to fight tumors [27]. The most promising results in pancreatic cancer treatment have come from direct targeting of the involved signaling molecules and immune checkpoint molecules, in combination with conventional therapies [27].

One of the main difficulties in the therapeutic management of patients with chronic pancreatitis is represented by the incorrect diagnosis of pancreatic cancer at an early stage. The differentiation between chronic pancreatitis and pancreatic cancer could be a challenge, leading to a delay in diagnosis and treatment [28]. One study that followed 471,992 subjects pointed out that approximately 5% of the patients included in the study group were initially misdiagnosed with chronic pancreatitis, and in two-thirds of them, the diagnosis of cancer was delayed by more than 2 months [28].

Taking into consideration the poor prognosis of patients with pancreatic cancer, mainly because of the late diagnosis in stages exceeding the therapeutic potential, we carried out a study to identify several clinical and paraclinical parameters that can contribute to the differential diagnosis between chronic pancreatitis and pancreatic cancer to avoid a delay in the diagnosis of pancreatic cancer. An early and accurate diagnosis of pancreatic cancer is crucial because it could raise the percentage of patients who are eligible for surgical treatment—the only potentially curative therapeutic method nowadays.

## 2. Materials and Methods

We conducted a retrospective, observational study on a sample of 120 patients diagnosed with pancreatic cancer or chronic pancreatitis (a representative sample for a population of patients diagnosed with pancreatic cancer or chronic pancreatitis) in a tertiary center for diagnosis and treatment.

Within the study, which was carried out over a period of 3 years in the Clinical Emergency Hospital of Bucharest, Romania, 120 patients who met the inclusion criteria were enrolled after signing the informed consent. The study was approved by the Ethics Committee of the Clinical Emergency Hospital of Bucharest (approval no 3929/12.04.2021).

The inclusion criteria were:

- Group A: patients with pancreatic cancer stages II–IV.
- Group B: patients with risk factors for pancreatic cancer.

The exclusion criteria were the existence of personal pathological history of cancer with another location or synchronous cancer at the time of enrollment, the lack of informed consent or patients whose medical documents had errors.

The collected data were centralized in Microsoft Excel. Furthermore, we used the R program for the statistical analysis with the following packages: effects, ggplot2, ggpubr, gtsummary, logisticRR [29,30,31,32,33,34,35].

The significance threshold for α was chosen to be 0.05, thus *p*-values lower than 0.05 were considered statistically significant. Gaussian distribution was verified using the Shapiro–Wilk test and quantile–quantile graphs (qq plots).

Two distinct directions have been followed during the study, as follows:

First research direction: identification of demographic and clinical variables which could be considered risk factors (predictors) for the occurrence of pancreatic cancer (the control sample being made up of patients diagnosed with chronic pancreatitis). The main endpoints of the study were the odds ratio (OR) values of the predictors of pancreatic cancer vs. chronic pancreatitis. The analysis consisted of a simple univariate binomial logistic regression, with the dependent variable represented by the condition of which the patient suffers (1 for pancreatic neoplasm, 0 for chronic pancreatitis). The independent variables were the variables followed in the study, whose predictive role was meant to be determined. Since the notion of odds does not have a counterpart in Romanian (the most acceptable translation being probability), and the notions of odds and OR are difficult to interpret in the medical scientific field, it has been decided to transform OR into the relative risk rate (RR, risk ratio), a much more familiar term in the medical field. It was not feasible to do this operation by using a direct calculation method, considering that relative frequencies (prevalence) of pancreatic cancer and chronic pancreatitis are not entirely known and, consequently, neither is the ratio between them. Hence, we decided to calculate the RR by using a bootstrap resampling methodology, with the RR value being equal to the median of the bootstrap distribution, while the 95% CI (95% confidence interval) was constructed using 2.5% and 97.5% quantiles of the bootstrap distribution. In addition, the effect of the predictor on the probability of pancreatic cancer was represented graphically.

Second research direction: as for the analysis procedure, a non-parametric test was used for the clinical and paraclinical variables that were continuous—a Wilcoxon bidirectional rank-sum test for two independent samples (also known in the literature as the Mann–Whitney U test) considering that the distributions of the variables showed significant deviations from a Gaussian distribution. A two-way Pearson χ2 (chi-square) test was also used for the categorical variables.

## 3. Results

We evaluated by comparison the influences of gender, age, body mass index (BMI), smoking, alcohol consumption, presence of personal pathological or heredo-collateral history (acute pancreatitis, diabetes, gallstones, cancer of another location) on the risk of pancreatic cancer vs. chronic pancreatitis.

**Figure 2:**
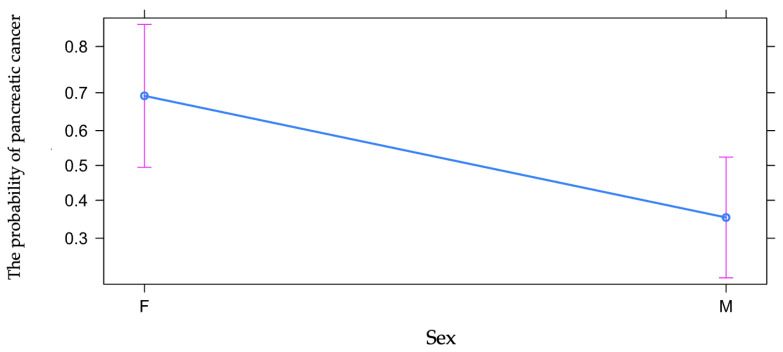
Predictive effect of gender on odds of pancreatic cancer vs. chronic pancreatitis.

The OR of gender influence on the risk of pancreatic cancer vs. chronic pancreatitis was 0.24, the effect being statistically significant. The relative risk rate was 0.51 (calculated with 10,000 bootstrap samples), with 95% CI 0.27 to 0.83, so that in the group under study the risk rate of pancreatic cancer vs. chronic pancreatitis was two times higher among women, by contrast, than men (Table 1 and Figure 2).

**Figure 3:**
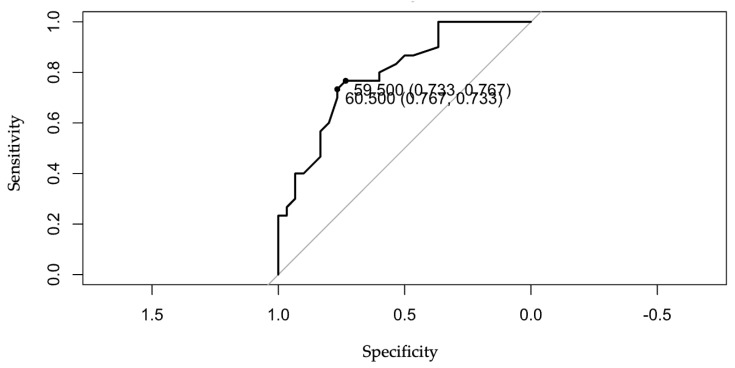
ROC analysis of pancreatic cancer vs. age.

A one-year increase in patient age seems to be associated with a 12% increase in the odds of pancreatic cancer vs. chronic pancreatitis (as this is a very small odds value, it equals the risk, so as OR = RR), the effect being statistically significant (Table 1). The age variable is a continuous variable. In order to calculate a cut-off value for age so as to maximize the probability of pancreatic cancer occurrence, an ROC analysis was used by means of the R pROC package (Table 1 and Figure 3) [30].

**Figure 4:**
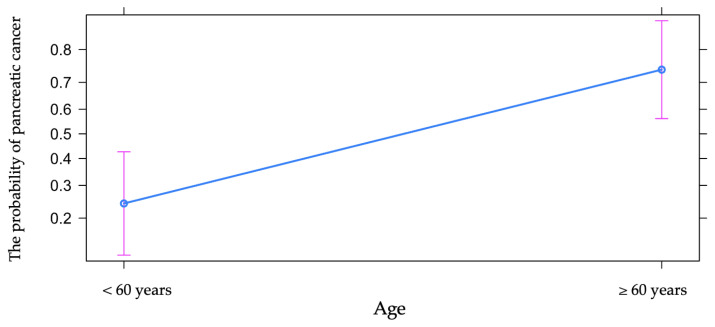
The influence of age on the risk of pancreatic cancer.

It is to be noted that there are two values for the cut-off, but since both are not present in our database (because the age variable was quantified using positive integers), we decided to use the value of 60 years as the cut-off value, dividing the sample of patients into two categories: older than or equal to 60 years old and younger than 60 years old. The odds of pancreatic cancer were nine times higher in patients older than or equal to 60 years, with the result showing statistical significance (Figure 4).

The relative risk rate was 3.07 (calculated with 10,000 bootstrap samples), with 95% CI 1.73 to 8.16. Thus, in the studied group, the risk rate of pancreatic cancer vs. chronic pancreatitis was three times higher for patients older than or equal to 60 years old compared to patients younger than 60 years old (Table 1).

Regarding BMI, the 10% increase in odds for 1 unit of BMI has no statistical significance, the values for RR being equal to those of OR (Table 1).

**Figure 5:**
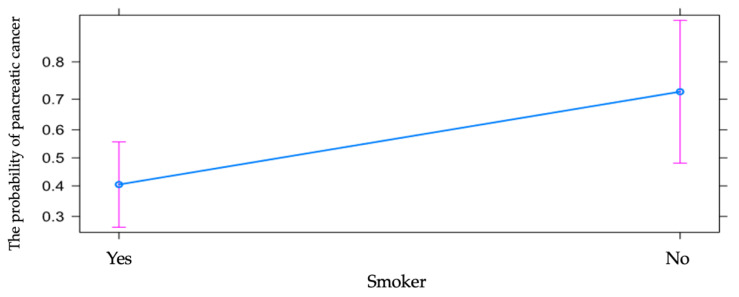
The influence of smoking on the risk of pancreatic cancer vs. chronic pancreatitis.

Even if smoking represents a considerable risk factor for the occurrence of pancreatic cancer, the odds of cancer vs. chronic pancreatitis was almost four times higher in non-smoking patients when compared to smoking or ex-smoking patients, with the effect being statistically significant. The relative risk ratio was 1.78 (calculated with 10,000 bootstrap samples), with 95% CI 1.09 to 3.00, so that in the studied group, the risk ratio of pancreatic cancer vs. chronic pancreatitis was 1.78 times higher among non-smoking patients vs. smoking patients (Table 1 and Figure 5).

**Figure 6:**
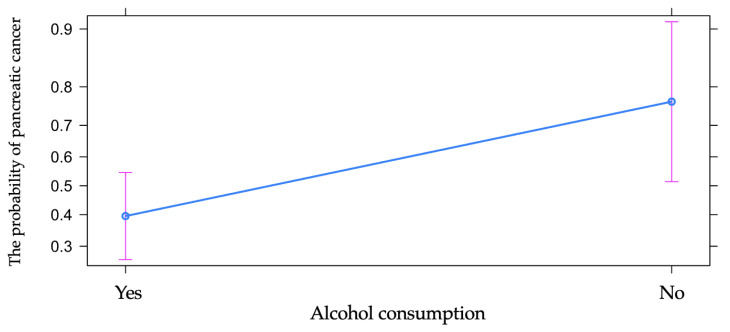
The influence of alcohol consumption on the risk of pancreatic cancer vs. chronic pancreatitis.

Alcohol consumption represents one of the most important risk factors involved in the occurrence of pancreatic cancer. However, the analysis by comparison showed the odds of cancer vs. chronic pancreatitis to be almost five times higher in patients who denied alcohol consumption when compared to patients who confirmed alcohol consumption, with the effect being statistically significant. The relative risk ratio was 1.93 (calculated with 10,000 bootstrap samples), with 95% CI 1.20 to 3.20, so that in the studied group, the risk ratio of pancreatic cancer vs. chronic pancreatitis was 1.93 times higher among patients who denied alcohol consumption vs. patients who affirmed chronic alcohol consumption (Table 1 and Figure 6).

Regarding the influence of history of acute pancreatitis, the odds of pancreatic cancer vs. chronic pancreatitis were 45 times higher in patients without a personal history of acute pancreatitis, with the effect being statistically significant. The relative risk ratio was 7.87 (calculated with 10,000 bootstrap samples), with 95% CI 3.44 to 83.70, so that in the studied group a history of acute pancreatitis was associated with a higher risk of evolution to chronic pancreatitis than to pancreatic cancer (Table 1).

Regarding the history of type 2 diabetes, the odds of pancreatic cancer vs. chronic pancreatitis were approximately two times lower in patients without a personal history of type 2 diabetes, but the effect had no statistical significance. The relative risk rate was 0.75 (calculated with 10,000 bootstrap samples), with 95% CI 0.45 to 1.30, so that in the studied group, the risk rate of pancreatic cancer vs. chronic pancreatitis was 25% lower among patients who did not have a history of type 2 diabetes when compared to patients with a history of type 2 diabetes (Table 1).

The odds of pancreatic cancer vs. chronic pancreatitis were 39% lower in patients without a hereditary history of cancer (HHC), but the effect was not statistically significant. The relative risk ratio was 0.79 (calculated with 10,000 bootstrap samples), with 95% CI 0.47 to 1.43, so that in the studied group, the risk ratio of pancreatic cancer vs. chronic pancreatitis was 21% lower in patients who did not have HHC vs. patients with HHC (Table 1).

The odds of pancreatic cancer vs. chronic pancreatitis were 37% higher in patients without hereditary history of type 2 diabetes, but the effect had no statistical significance. The relative risk ratio was 1.17 (calculated with 10,000 bootstrap samples), with 95% CI 0.67 to 2.59, so that in the studied group, the risk ratio of pancreatic cancer vs. chronic pancreatitis was 17% higher in patients who did not have a hereditary history of type 2 diabetes vs. patients with hereditary history of type 2 diabetes (Table 1).

In the second part of the study, a series of biological paraclinical variables were compared between the two study groups. The tests for serum hemoglobin values, leukocyte count, platelet count and serum glucose values did not identify statistically significant differences between the two groups of patients.

**Figure 7:**
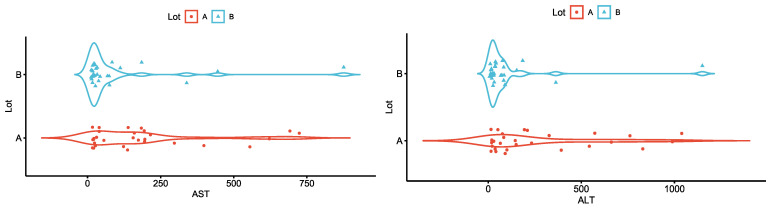
Median AST and ALT values in patients with pancreatic cancer (group A) vs. chronic pancreatitis (group B).

The comparative analysis between the two study groups regarding the serum values of aspartate aminotransferase (AST) and alanine aminotransferase (ALT) identified statistically significant differences. Thus, median AST was approximately five times higher in patients with pancreatic cancer than in patients with chronic pancreatitis, and median ALT was approximately three times higher in patients with pancreatic cancer (Figure 7).

**Figure 8:**
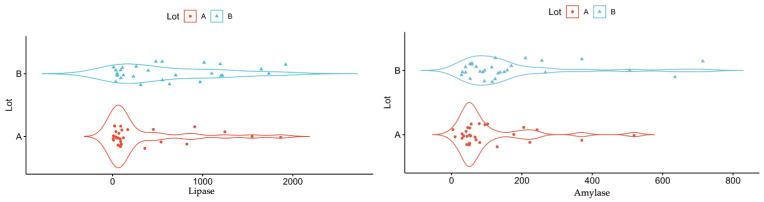
Median values of serum lipase and serum amylase in patients with pancreatic cancer vs. chronic pancreatitis.

Concerning the serum values of lipase, amylase, total bilirubin, direct bilirubin and alkaline phosphatase, the comparative analysis between the two study groups identified statistically significant differences. Thus, in patients with pancreatic cancer, the median value of serum lipase was almost five times lower than in patients with chronic pancreatitis. Moreover, the median value of serum amylase is more than two times lower in patients with pancreatic cancer when compared to the group with chronic pancreatitis (Table 2 and Figure 8).

In patients with pancreatic cancer, total bilirubin values were four times higher than in the group with chronic pancreatitis, and direct bilirubin values were seven times higher. For gamma-glutamyl transferase (GGT) values, no statistically significant differences were identified between the two study groups, but the median value of alkaline phosphatase in cancer patients was almost three times higher than the median value in patients with chronic pancreatitis (Table 2).

By evaluating the serum values of total cholesterol and triglycerides, the statistical analysis of the lipid profile did not identify statistically significant differences between the two groups of patients (Table 2).

**Figure 9:**
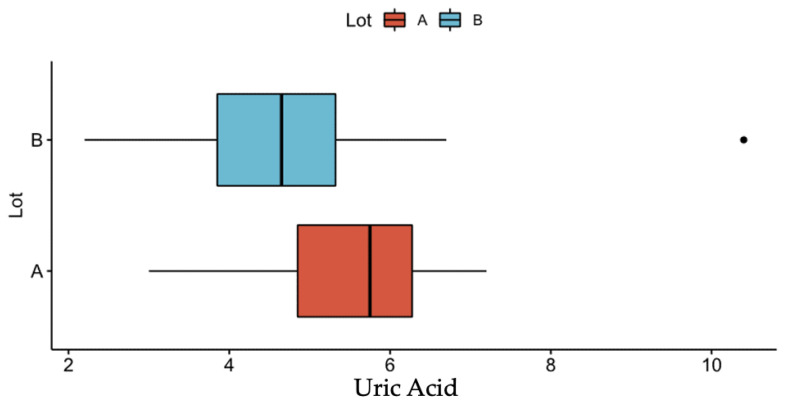
Median serum uric acid value in patients with pancreatic cancer (group A) vs. patients with chronic pancreatitis (group B).

Regarding the inflammatory markers, serum C-reactive protein (CRP) and erythrocyte sedimentation rate (ESR) values were evaluated. While concerning the values of ESR there were no statistically significant differences between the two groups of patients, when it comes to CRP, the median value in patients with pancreatic cancer exceeded almost two times the median value of CRP among patients with chronic pancreatitis (Table 2). Furthermore, regarding uric acid, the statistical analysis pointed out an increase in the median blood value by 1.10 mg in patients with pancreatic cancer compared to the group with chronic pancreatitis, with this difference being statistically significant (Table 2 and Figure 9).

Another category of biological markers evaluated are represented by the tumor markers CEA and CA19-9. Considering that a small proportion of patients benefited from the dosing of these markers (for CEA—24 patients, and for CA19-9—28 patients), the statistical analysis identified a marginally insignificant *p*-value in both cases. Therefore, in patients with pancreatic cancer, the median values of CEA were two times higher compared to patients with chronic pancreatitis, and median values of CA19-9 were more than 70 times higher compared to patients with chronic pancreatitis.

## 4. Discussion

Pancreatic cancer has a high rate of mortality. The negative prognosis is mainly the result of late diagnosis, usually in stages that cannot allow surgical treatment with a curative aim. One of the reasons for late diagnosis is represented by the nonspecific symptoms up to advanced stages of the disease. The initial clinical presentation of patients with pancreatic cancer varies based on the location of the primary tumor. The tumor is localized in the head of the pancreas in approximately 60–70% of the cases, in the body or tail of the pancreas in 20–25% of the cases, and in approximately 5–20% of the cases the tumor affects the whole pancreas [36].

The three most frequent symptoms of patients with pancreatic cancer are abdominal pain, jaundice and weight loss [36]. Those patients with the tumor localized in the head of the pancreas have particular symptoms. They usually present with jaundice, weight loss and steatorrhea [36,37]. If the tumor is located in the head of the pancreas, jaundice appears in an early stage and plays a role in prognosis. Thus, the patients that present with jaundice and abdominal pain have a worse prognosis compared to patients who present with jaundice but no abdominal pain. Jaundice may also appear when the tumor is located in the body or the tail of the pancreas; however, it appears relatively later due to the extension of the primary tumor or the appearance of hepatic metastases.

Even though weight loss and steatorrhea are manifestations of exocrine pancreatic insufficiency, they are also met in patients with chronic pancreatitis [28]. There are reports in the literature that recognize the possibility of an erroneous diagnosis between pancreatic cancer and chronic pancreatitis [28]. This leads to a delay in the diagnosis of pancreatic cancer that is biologically aggressive and, secondarily, to negative consequences upon the prognosis of the patients [28]. The exact proportion of patients with pancreatic cancer wrongly diagnosed with chronic pancreatitis is not known due to limited data. An estimate of 5% is given by Munigala et al. [28].

The analysis of data obtained in our study led to statistically significant results. Thus, a comparative analysis showed that the relative risk rate of pancreatic cancer vs. chronic pancreatitis is two times higher in women compared to men (95% CI 0.27–0.83). Furthermore, an increase in the age of the patient by 1 year was shown to have led to an increase in the odds of pancreatic cancer with 12% (95% CI 2.94 to 31.2). These results are consistent with others from the specialized literature. Wang et al. reported a risk ratio of chronic pancreatitis in men vs. women of 4.5 [38]. Studies on animals proved the implications of estradiol in the pathogenesis of chronic pancreatitis. Thus, treatment with estradiol seems to attenuate the apoptosis of acinar cells independently of factors such as the modifications mediated by T cells or the levels of corticosterone or testosterone [38]. However, a study on mice demonstrated that only male mice can spontaneously develop chronic pancreatitis [39]. Other studies on animal models and human tissues show the role of reactive oxygen species (ROS) in the pathogenesis of chronic pancreatitis, together with a variable capacity to reduce the levels of ROS based on gender [40]. Male mice proved to have higher levels of ROS but also more important inflammatory and fibrotic modifications of the pancreas compared to female mice [40]. Even though the specialized literature associated the male gender with a higher risk of pancreatic cancer, in our study men had a higher risk of chronic pancreatitis. Banerjee et al. suggest that methoxyestradiol has a biphasic effect on pancreatic oncogenesis [41]. Thus, high doses of methoxyestradiol determine an increase in the vascular level of endothelial growth factor A (VEGF-A) and the stimulation of cellular proliferation, while low doses have the exact opposite effects [41]. Furthermore, Skipworth et al. highlight that the prognosis of patients with pancreatic adenocarcinoma is more unfavorable for men with low levels of testosterone and women with high levels of estrogen [42]. In terms of age, this type of neoplasia is well-known to affect more frequently the elderly patients [43]. The average age at the diagnosis of pancreatic cancer is approximately 72 years [44]. By contrast, the average age of patients with chronic pancreatitis is significantly lower, in the range of 35–55 years [45]. Thus, our study validates other data reported in the literature.

While observing the BMI values, our results were not statistically significant. One possible explanation is that for most of the patients in our study, the BMI was evaluated after a significant weight loss secondary to the syndrome of neoplastic impregnation.

Alcohol consumption and smoking were associated with chronic pancreatitis rather than pancreatic cancer. In studies on animal models, exposure to cigarette smoke for several weeks led to pancreatic lesions and increased levels of digestive zymogens, chymotrypsinogen and trypsinogen [46,47]. Furthermore, these mice also showed modifications of gene expression and the ratio between the endogen inhibitor of trypsinogen and the trypsinogen [46]. Askari et al. highlight the increase in the toxicity of alcohol due to smoking in patients with pancreatitis [47]. These authors emphasize the aggravation of ischemic alterations and the increase of leukocyte infiltration of the pancreas when combining alcohol consumption with smoking [48]. Edderkaoui et al. concluded that smoking increases not only the risk of chronic pancreatitis but also the risk of evolution of chronic pancreatitis to pancreatic cancer [47]. The data on the role of alcohol consumption in the development of pancreatic cancer are contradictory. A detailed analysis of the studies that reported a correlation between this risk factor and pancreatic cancer showed an association of smoking with alcohol consumption [49,50]. Nevertheless, a weak or false correlation between alcohol and pancreatic cancer cannot be excluded [49,50]. However, alcohol consumption is a well-known risk factor for chronic pancreatitis, which, in turn, is a risk factor for pancreatic cancer [49,50]. In conclusion, our study supports the etiopathogenetic sequence smoking/alcohol consumption–chronic pancreatitis–pancreatic cancer.

Our study identified a single statistically significant correlation related to personal pathologic and heredo-collateral pathological history. The correlation was found in the personal history of acute pancreatitis and the evolution towards chronic pancreatitis (*p* < 0.001). This result is validated by other studies which support the etiopathogenetic correlation [51,52]. A study that followed 352 patients with a history of acute pancreatitis over 30 years reported a progression rate towards chronic pancreatitis of 24.1% [53]. The average duration before the diagnosis of chronic pancreatitis in patients with at least one episode of acute pancreatitis was 3.5 years [53].

In the second part of our study, we made a comparative analysis between the two groups of a series of biological parameters from the peripheric blood. As a result, the patients with pancreatic cancer had higher values of serum aminotransferases, while the patients with chronic pancreatitis had higher values of serum amylase and lipase. The presence of liver metastases may explain the hepatic cytolysis syndrome in patients with pancreatic cancer. It is well known that the liver is the most frequent location for metastases from pancreatic cancer [54]. Furthermore, some data support the hypothesis that the process that leads to hepatic metastases is initiated early in the evolution of the pancreatic adenocarcinoma, possibly even in the premalignant phases [54,55]. A series of factors secreted by the pancreatic tumoral cells, especially the exosomes, tissue factor (TF) and tissue inhibitor metalloproteinase-1 (TIMP-1), contribute to the transformation of the liver into a favorable niche [54,55,56].

The elevated levels of serum amylase and lipase in patients with chronic pancreatitis may be explained through chronic inflammatory changes in the pancreas. A study comparing patients with chronic pancreatitis with patients with pancreatic cancer identified increased values of these enzymes in 40% of the patients with chronic pancreatitis and 0% of those with cancer (*p* = 0.002) [57]. Weiss et al. emphasized a genetic predisposition for chronic pancreatitis in individuals with fucosyltransferase 2(FUT2) non-secretor status and those with blood group B [58]. Even in the absence of symptoms in these patients, elevated levels of lipase can indicate the presence of subclinical pancreatic lesions [58].

Another syndrome that we found to be present in patients with pancreatic cancer is the cholestasis syndrome. Two possible reasons are the compression of the main biliary duct by the pancreatic tumor or the presence of hepatic metastases. Cardillo et al. evaluated a group of 123 patients with pancreatic cancer and identified the clinical manifestations of the icteric syndrome in 37% of the patients [59]. The cause of the jaundice was biliary obstruction, cholangitis or hepatic metastases [59]. The local development of pancreatic tumors is one of the determinant factors of the mortality rate [59]. Iacobuzio-Donahue et al. identified during autopsy that the local development of the tumor was the main cause of death in 30% of the patients with pancreatic cancer [60].

We evaluated the systemic inflammatory syndrome through the values of the CRP and found it to be more elevated in patients with pancreatic cancer compared to those with chronic pancreatitis. Recent data proved the role of systemic inflammatory response (SIR) in the pathogenesis of several types of cancers, including pancreatic ductal adenocarcinoma [61]. SIR proved to be a promoter of the proliferation, invasion and metastasis of tumoral cells [62,63,64]. A study on 419 patients with advanced pancreatic cancer reported the utility of certain markers of systemic inflammation in the prognosis of these patients [65]. Furthermore, the authors emphasize that the prognostic value of inflammatory markers is independent of the values of other prognostic markers such as CA19-9 [65]. Nurmi et al. validated the use of a combined score based on CRP and CA19-9 in the presurgical prognostic evaluation of patients with pancreatic cancer [66]. In this study, even a small increase in the CRP values had a significant impact on the prognosis of patients with pancreatic cancer [66]. Among patients with chronic pancreatitis, this inflammatory marker is a sign of the inflammatory status of the pancreas, thus increasing only in the acutization periods of the disease [67,68]. In conclusion, our study validates other data from the literature, distinguishing CRP as a marker that can help the differential diagnosis of the two diseases considered here.

According to recent data, the serum level of uric acid correlates with the serum level of certain inflammatory markers that act as promoters of oncogenesis [69,70]. Some elements of the inflammatory microenvironment with a demonstrated role in oncogenesis, such as CRP, cyclooxygenase 2, adiponectin and leptin, were proved to corelate with the levels of uric acid [69,71]. Xie et al. concluded in a metanalysis from 2019 that the elevated level of uric acid increases the risk of cancer, no matter the location of the cancer [72]. Another study published in 2020 identified a link between the elevated level of uric acid and the risk to develop pancreatic cancer in women or gallbladder cancer in men [73]. In our study, the levels of uric acid were significantly higher in the patients with pancreatic cancer compared to those with chronic pancreatitis. Furthermore, the comparative analysis between the two diseases resulted in twice the frequency of pancreatic cancer in women compared to men. Thus, we can indirectly validate the link between the elevated level of uric acid and the risk of pancreatic cancer in female patients.

CEA and CA 19-9 are biological markers that were proven to be significantly increased in patients with pancreatic cancer compared to patients with chronic pancreatitis. Our analysis was not statistically significant because of the low number of patients in whom the two markers were evaluated. The only biomarker currently used in the management of patients with pancreatic cancer is the carbohydrate antigen 19-9 (CA19-9) [74]. CA19-9 was proven to play a role in the prognosis rather than the diagnosis of a patient as the positive predictive capacity was 57.1% in patients with pancreatic cancer in stage I and 44.1% in those in stage II [11]. Thus, this biomarker may be used for patients who are considered to suffer from pancreatic cancer, either due to pathological modifications found by imaging or the presence of any suggestive symptoms [11]. Furthermore, CA19-9 may be used for monitoring the recurrence of the tumor after treatment [11]. However, Lee et al. not only proved the role in prognosis of CA19-19 but also of CEA for the patients with pancreatic adenocarcinoma, especially those patients who do not benefit from surgical treatment [75].

The main limitations of our study are the relatively small number of patients included in the analysis and the inability to test the tumoral markers for the entire group of patients. The peculiarity of our study consists in the specific comparative analysis between patients with pancreatic cancer vs. chronic pancreatitis who presented themselves in an emergency hospital. This aspect can explain the size of the study group. However, our study identified a series of clinical and biological data that can be evaluated from the first visit to the doctor and can later guide the diagnostic management.

Additionally, for the analysis of continuous clinical and paraclinical variables, the Wilcoxon bidirectional rank-sum test was used, with a lower power compared to a parametric tests. Hence, future studies are necessary on larger cohorts of patients, to identify clinical and paraclinical data that can further contribute to the differential diagnosis of the two diseases considered here. Given the low incidence of pancreatic cancer and chronic pancreatitis, our goal was to identify certain biomarkers that are commonly tested, to preserve the balance between cost and efficiency.

## 5. Conclusions

The comparative analysis of patients with pancreatic cancer vs. chronic pancreatitis identified female gender and advanced age to be more frequently associated with pancreatic cancer. Patients with chronic pancreatitis have more frequently history of acute pancreatitis. Regarding biological data, the patients with chronic pancreatitis showed higher serum values of amylase and lipase, while patients with pancreatic cancer had higher values of aminotransferases, cholestasis markers, CRP and uric acid. The analysis of these laboratory data may guide the diagnosis towards chronic pancreatitis or pancreatic cancer, allowing an earlier diagnosis and improving the prognosis of these patients.

## Figures and Tables

**Table 1:** Epidemiological and clinical parameters in patients with pancreatic cancer vs. chronic pancreatitis.

Predictor	N	Chronic Pancreatitis (n = 60)	Pancreatic Cancer (n = 60)	OR (95% CI)	*p*
Sex	120				
Female		16	36	-	
Male		44	24	0.24 (0.08, 0.70)	0.011
Age	120			1.12 (1.06, 1.21)	<0.001
Age groups	120				
<60 years		44	14	-	
≥60 years		16	46	9.04 (2.94, 31.2)	<0.001
BMI	120	60	60	1.10 (0.98, 1.25)	0.111
Smoker	120				
Yes		50	34	-	
No		10	26	3.82 (1.20, 13.8)	0.029
Alcohol consumption	120				
Yes		52	34	-	
No		8	26	4.97 (1.48, 20.0)	0.014
History of acute pancreatitis	120				
Yes		50	6	-	
No		10	54	45.0 (11.1, 251)	<0.001
History of diabetes mellitus type II	120				
Yes		44	24	-	
No		16	36	0.55 (0.18, 1.60)	0.276
History of biliary lithiasis	120				
Yes		40	18	-	
No		20	42	1.17 (0.39, 3.52)	0.781
Familial history of diabetes mellitus type II	120				
Yes		20	16	-	
No		40	44	1.37 (0.45, 4.270)	0.574
Familial history of cancers	120				
Yes		14	20	-	
No		46	40	0.61 (0.19, 1.88)	0.392
	OR = odds ratio; CI = confidence interval; BMI = body mass index.				

**Table 2:** Biological parameters in patients with pancreatic cancer vs. chronic pancreatitis.

Biological Parameters	N	Group A; N = 60	Group B; N = 60	*p* ^1^
Hemoglobin, Median (IQR)	120	12.35 (11.55–13.38)	12.80 (11.68–13.88)	0.859
Leukocytes, Median (IQR)	120	7.660 (6.322–9.608)	7.635 (6.240–9.925)	0.824
Platelets, Median (IQR)	120	241.000 (202.000–298.000)	294.000 (225.000–366.000)	0.099
Glycemia, Median (IQR)	120	117 (101–134)	102 (92–124)	0.322
Total cholesterol, Median (IQR)	120	173 (134–214)	168 (149–184)	0.959
Triglycerides, Median (IQR)	120	135 (111–193)	120 (84–148)	0.072
Aspartate aminotransferase (AST), Median (IQR)	120	139 (27–196)	26 (18–73)	0.005
Alanine aminotransferase (ALT), Median (IQR)	120	100 (37–392)	34 (19–82)	0.006
Lipase, Median (IQR)	120	88 (46–358)	395 (89–1015)	0.029
Amylase, Median (IQR)	120	55 (44–101)	116 (58–170)	0.020
Total bilirubin, Median (IQR)	120	4 (1–16)	1 (0–1)	<0.001
Direct bilirubin, Median (IQR)	120	3 (0.40–12)	0.40 (0.20–1)	<0.001
Gamma-glutamyl transferase (GGT), Median (IQR)	120	188 (106–806)	116 (46–359)	0.078
Alkaline phosphatase, Median (IQR)	120	348 (149–518)	132 (78–268)	0.030
C-reactive protein, Median (IQR)	120	33 (23–42)	17 (7–39)	0.049
Erythrocyte sedimentation rate, Median (IQR)	120	34 (28–40)	29 (21–57)	0.830
Uric acid, Median (IQR)	120	5.75 (4.85–6.27)	4.65 (3.85–5.32)	0.001
Carcinoembryonic antigen (CEA), Median (IQR)	24	4 (2–20)	2 (1–2)	0.073
Carbohydrate antigen 19-9 (CA 19-9), Median (IQR)	28	849 (55–950)	12 (8–57)	0.096

^1^ Wilcoxon rank sum test.

## Data Availability

Not applicable.
